# Heart Transplantation: New Decade, New Perspectives

**DOI:** 10.21470/1678-9741-2020-0601

**Published:** 2020

**Authors:** Jessica A. Steadman, Richard C. Daly

**Affiliations:** 1 Department of Cardiovascular Surgery, Mayo Clinic 200 First Street, SW, Rochester, MN, United States of America

Despite advances in medical and mechanical therapy, outcomes for end-stage heart failure patients remain poor. For a carefully selected cohort, heart transplantation remains the treatment of choice. Given limited organ availability, heart transplantation warrants careful consideration of the risk and benefits of transplant as well as a patient’s individual capacity to benefit from the transplant relative to the other pool of candidates. Heart transplantation presents an area of ongoing research and innovation aimed at solving the key limitations to transplantation, namely organ shortage and post-transplant allograft rejection.

Traditionally, cardiac allograft procurement occurs following donation after brainstem-determined death (DBD). Advantages of DBD include the ability to assess the organ prior to procurement allowing for optimal organ selection, controlled cardiac arrest and protection with cold cardioplegia, and avoidance of the detrimental effects of warm ischemia^[1]^. Since 2015, several centers have used hearts obtained following donation from select donors after circulatory-determined death (DCD)^[2]^. A study from the United Kingdom reported similar allograft performance and 90-day survival rates following DCD heart transplantation as compared to DBD transplantation^[1]^. These promising results offer a potential solution to bridge the gap between organ demand and availability. Forecasts have predicted that DCD heart transplantation could increase transplantation rates by up-to 20%^[3]^. Over the coming decade, we envisage more wide-spread use of DCD transplantation with greater standardization of procurement protocols to ensure organ quality and protection.

*Ex-vivo* perfusion of donor organs is another potential method for expanding donor heart availability. By preserving organs for longer time thus enabling transport to more distant locations, the use of such systems can allow for more even distribution of organs across regions. Moreover, donor hearts can be resuscitated, reducing the number of organs considered unsuitable for transplant^[4]^. The PROCEED II trial showed *ex-vivo* perfusion using the Organ Care System® (TransMedics, Andover, Massachusetts, United States of America) to be equivalent to cold ischemic preservation and with no detrimental effect on short-term patient or graft outcomes^[4]^. In addition, *ex-vivo* machine perfusion in the context of DCD donation could allow reanimation of the heart and restoration to near physiological state prior to transplantation^[5]^. Starting this year, the Mayo Clinic Transplant Center is launching a lung restoration center with the goal of increasing the availability for transplantation using *ex-vivo* lung perfusion. Optimized lungs will then be available to several transplant centers across the United States of America. With the improvement in *ex-vivo* perfusion technology, we envisage that a similar concept for heart preservation could be developed.

Allosensitization to human leukocyte antigens (HLA) remains one of the biggest impediments to safe heart transplantation. It reduces access to otherwise ABO-compatible donors thus increasing transplant waiting time^[6]^. Transplanting across a positive crossmatch is avoided due to the associated high risk of early graft rejection and poorer long-term outcomes from higher rates of coronary allograft vasculopathy^[7]^. However, in select, sensitized patients, de-sensitization protocols have been trialed to increase the chances of a negative crossmatch and improve transplant outcomes. Plasmapheresis and intravenous immunoglobulins have historically been the mainstay for de-sensitization treatments, aimed at removing currently circulating antibodies. More recently, monoclonal antibodies targeting specific cells of the immune system have been paired with these. Rituximab, which targets CD20 on B cells, aims to reduce the production of new antibodies. Similarly, Bortezumab, targeting plasma cells, inhibits antibody production but is more targeted towards anti-HLA I antibodies. Finally, Eculizumab targets the complement pathway and is currently being investigated for the treatment of sensitized heart transplant candidates in the DUET Cardiac Trial^[8]^.

Multi-organ transplantation involving the liver offers a unique opportunity to address pre-transplant sensitization. Liver allografts alone can decrease donor-specific antibodies (DSA) levels. Mayo Clinic reported the first experience of performing combined heart-liver transplantation with implantation of the liver prior to the heart allograft. The aim was to protect the heart from antibody-mediated rejection (AMR). This technique successfully reduced DSA levels immediately after the liver transplant in all patients and resulted in normal cardiac allograft function, and only one case of AMR was recorded post-transplant^[9]^.

A final area of ongoing research in the context of heart transplantation focuses on detection of graft rejection. Traditionally, repeated endomyocardial biopsies are assessed for histological evidence of rejection. However, these procedures are associated with considerable morbidity over the long term such as catheter-related complications, tricuspid regurgitation, and renal insufficiency. In a select recipient population at low risk of rejection, gene expression profiling has reduced biopsy numbers without increased risk of serious adverse outcomes^[10]^. Alternatively, measurement of donor-specific cell-free deoxyribonucleic acid (DNA) has been proposed as a marker for cellular injury caused by rejection^[11]^. With a growing body of evidence to support less invasive methods of monitoring cardiac graft rejection, we hope to see a reduction in the need for invasive endomyocardial biopsies in the future.

While clearly conferring a survival benefit, heart transplantation is currently only available to a select cohort of patients. In addition, long-term morbidity and limited graft longevity renders transplantation a treatment rather than cure for heart failure. The ongoing developments promise to contribute to improve both organ availability and graft longevity over the coming decade.
